# Percutaneous transhepatic biliary puncture simulator: a cord network prototype

**DOI:** 10.1186/s41077-021-00178-w

**Published:** 2021-08-06

**Authors:** Rubén Lopez Benítez, Tomás Reyes del Castillo, David Benz, Carsten Fechner, Lorant Szabo, Levent Kara, Etienne Monnard, Michael Kostrzewa, Justus E. Roos

**Affiliations:** 1 Department of Radiology and Nuclear Medicine, Luzerner Kantonsspital, 6000 Lucerne, Switzerland; 2 Institute of Radiology and Nuclear Medicine, Stadtspital Triemli Zürich, 8063 Zurich, Switzerland; 3 Department of Radiology, Fribourg Hopital Cantonal, 1752 Fribourg, Switzerland; 4 Institute of Radiology, Kantosspital Baden, 5404 Baden, Switzerland

**Keywords:** Biliary puncture, Biliary tree, Bile duct, Interventional radiology, Simulation, Training, Education, Liver, PTC

## Abstract

**Background:**

The aim of this study was to present a percutaneous transhepatic biliary puncture simulator that can be used without radiation exposure and that reflects the conventional anatomy of the biliary ducts and its vicinity structures.

**Methods:**

An anatomically based model of the biliary tree was developed using a cord network fixed to a wooden frame. The skin, ribs, intercostal muscles, and right lower lobe pleura were simulated using foam sponge, plastic tubes, a polystyrene foam panel, and an air pad, respectively. For the puncture, we used a 20-G Chiba needle and a wire with distal double arches; these were used to troll a cord, simulating the successful puncture of a bile duct. A camera was also placed above the model to allow the trainees to train eye-hand coordination while viewing the image on a monitor in real time. The simulator was tested with 60 radiology residents to evaluate the confidence and skills transferability of the training model.

**Results:**

After receiving an introduction of the system and 5 min of training under tutor surveillance, all participants were able to troll a cord of the biliary simulator by themselves in less than 4 min. Only one participant punctured the simulated pleura. The participants’ evaluations showed positive results, with increased user confidence and skills transferability after the training session.

**Conclusions:**

This proposed simulator can be an effective tool to improve a trainee’s confidence and competence while achieving procedural and non-procedural interventional radiology skills related to the liver.

**Trial registration:**

Retrospectively registered

## Background

Performing a percutaneous transhepatic cholangiography (PTC) has several challenges and risks. The first step to successful PTC is to establish a proper percutaneous access into the biliary tree, and this usually requires sonographic and fluoroscopic guidance for real-time visualization of the needle and the guidewire manipulation [1]. Percutaneous biliary access is associated with complication rates that range from 3 to 10% and procedural mortality rates that range from 0.1 to 0.8%. The complications include pain, pleural transgression, pneumothorax, subcapsular hematoma, inadvertent arterial access, and bile duct perforation [2].

The first steps to establish a percutaneous access to the biliary tree consist of identifying the 9th or the 10th intercostal spaces and inserting a Chiba needle on the mid-axillary line through the skin and soft tissues. Subsequently, using fluoroscopy visualization, the needle is advanced through the liver with an orientation that goes from caudal to cranial and dorsal to ventral to puncture a right central branch; this maneuver should be performed with care to avoid an injury to the adjacent pleura. When in the desired position, the interventionist may suspect that the tip is inside a biliary duct if he feels a small resistance when advancing the needle; to corroborate this, biliary material is aspirated to further inject contrast medium, visualize the bile duct, and introduce a 0.018 stainless steel guide for cannulation. If the needle fails to puncture a biliary duct, the needle is withdrawn, and the puncture reattempted with a new orientation until the target is achieved [1, 2].

Most of the interventional radiology (IR) procedures used today are taught through the apprenticeship model. However, even under the best tutelage, this has some disadvantages, including prolonged intervention times, increased patients and operator X-ray doses, greater chance for errors, and unnecessary stress on the person who is learning [3]. In addition, most of these procedures are performed with the patient under only moderate sedation, so the patients themselves may realize that they are being used as subjects for training. Simulator models can help alleviate these limitations by creating opportunities to gain clinical and procedural skills while promoting patient safety [4]. In addition, feedback and repeated training can be performed almost unlimitedly until proficiencies are met [5]. If accurate simulators of IR procedures are developed and accepted, they could be used to formulate a standardized curriculum and shift the educational paradigm to a hybrid form of learning [6].

To our knowledge, there is no standard PTC simulator yet, although there is a report of a virtual reality system for PTCD simulation by Dirk Formeier et al. using haptic devices and virtual ultrasound and X-ray simulation. However, this simulator is not reproducible; it seems difficult to use and is not available on the market. From our perspective, understanding the three-dimensional orientation of the biliary branches and acquiring needle orientation and wire manipulation skills without using complex navigation systems is practical and achievable.

The aim of this study was to present a percutaneous transhepatic biliary puncture simulator that can be used without radiation exposure and that reflects the conventional anatomy of biliary ducts and its vicinity structures. This paper concentrates on a spatial orientation framework for the training of the first steps of PTC regarding needle navigation

## Methods

### Construction of the prototype

A cord network system attached to a wooden cube frame (30 × 25 cm) was developed according to the anatomical segmentation of the biliary tree proposed by Couinaud (eight independent functional segments) [7].

**Fig. 1:**
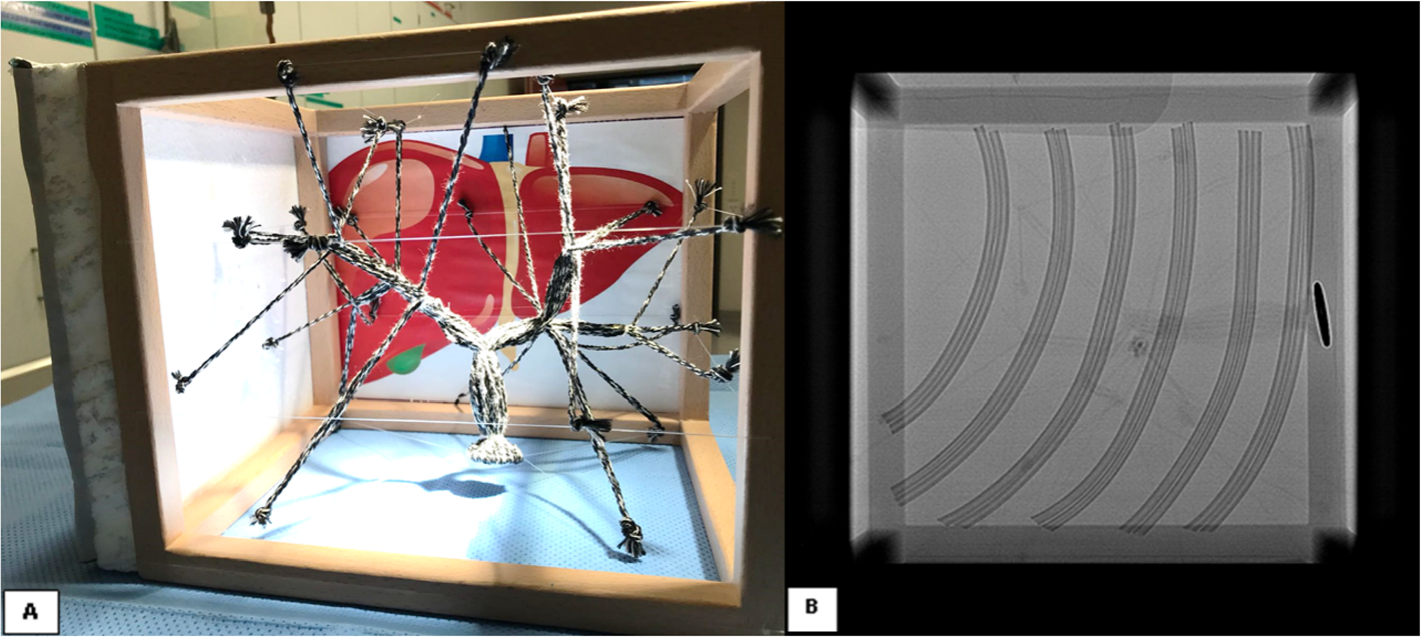
**a** Cord network system with the anatomical configuration of a biliary tree. **b** Fluoroscopic image demonstrating the ribs and the position used for simulation of the liver puncture

To simulate the natural decrease in the diameter of the bile ducts from the central to peripheral ducts, we bound a bundle of cords (1.8 mm nylon/cotton) to a metal ring at the caudal portion of the cube (simulating the main hepatic duct). We tied the first bifurcation a few centimeters down to simulate the left and the right biliary duct, and we continued the bifurcations into the periphery, following the anatomy of the liver segments and reducing the number of cords after every bifurcation. On the right side of the system, we placed a 2-cm-thick polystyrene foam (Styrofoam™) panel to simulate the intercostal muscles and subcutaneous tissue. To simulate the ribs, we fixed six bent polyurethane tubes on top of the polystyrene foam panel and covered them with a thin layer of foam sponge to simulate the skin (Fig. 1). This allowed the participants to palpate the intercostal spaces before puncturing the liver. The right pleura and the right costodiaphragmatic angle were simulated by the placement of an industrial air pad (5 × 8 cm) that could be perforated by the puncture needle while training.

**Fig. 2:**
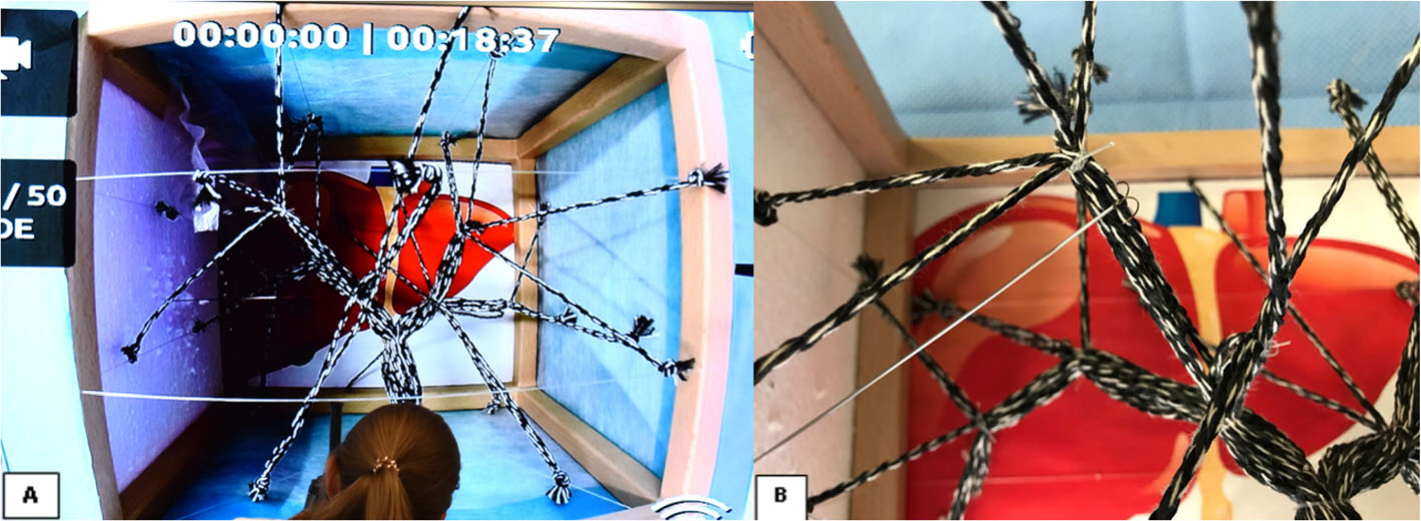
**a** Photography of the monitor during a simulation session. This is what the participant sees in real time. **b** Double-arch marking wire trolling a cord

For the puncture, we used 20-G Chiba needles. Over the needle, we introduced a twisted nitinol double wire with distal double arches (Somatex® Berlin, Germany) to troll a cord, thereby simulating the successful puncture of a bile duct (Fig. 2). To train eye-hand coordination under fluoroscopic interventions, we added a camera (GoPro 3®, USA), which was situated above the model, and broadcasted the image to a monitor to simulate the real-time visualization of the needle trajectory. Alternatively, the use of a smartphone camera is possible.

#### Content expert’s evaluation

The assessment of the simulator was done by a group of seven interventional radiology experts (RLB, TR, DB, LS, LK, EM, and MK), and consisted of evaluating the physical resemblance and functional task alignment of the simulator to train the first steps of PTC.

#### Physical resemblance

The simulator presents an acceptable physical resemblance to the right hypochondriac region of a patient in a supine position. The skin is dark and monochromatic, which makes the differentiation between the ribs and intercostal spaces difficult with the naked eye. The model has five palpable intercostal spaces (7th to 12th) and a soft tissue with a thickness of 2 cm. In addition, the cord network has anatomical coherence with a dilated intrahepatic biliary tract representing eight segments with branch diameters ranging from 4 to 9 mm (distal to central). The simulated pleura of the right costodiaphragmatic angle is located on the anterosuperior aspect of the wooden frame, which is like the anatomical position.

#### Functional task alignment

The simulator response to the clinical task of palpating and recognizing the intercostal spaces to select the skin entry point is realistic, the advancement of the needle through the soft tissue and into the liver is adequate. The visualization of the biliary branches in the monitor is not realistic but allows the participant to have anatomical reference and to be able to plan and aim his puncture. If the participant fails to puncture a biliary branch, the tip of the needle is compared with respect to the desired branch on the monitor, the needle is withdrawn, and the puncture reattempted with a new orientation; these steps are very realistic and allows the participant to learn how to deal with 3D space orientation in a 2D image. If a biliary branch was successfully punctured, the double arc guidewire was advanced with rotation maneuvers to troll a cord and mimic a successful cannulation of the biliary tree.

A demonstrative video is available on the following link: https://www.youtube.com/watch?v=MWkC-IiSe4Q&t=12s.

#### User’s evaluation

**Fig. 3:**
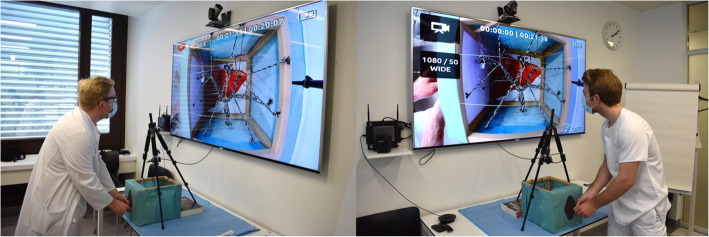
Photographs of the simulation tutorial showing the relationship between the PTC model, the camera, the monitor, and the participant

We tested our simulator on sixty participants (26 female, 34 male) from four different hospitals with radiology residence programs in Switzerland (Fig. 3). None of the participants had any previous experience with IR. Every participant received a tutelary introduction to the system and was given the opportunity to use the simulator multiple times during 5 min to reach the same level of competency. On a second round, we then measured the time it took each participant to troll a cord on their own. A 4-point Likert scale questionnaire was also handed out to every participant for the evaluation of the PTC simulator. The questionnaire consisted of eight questions and evaluated six aspects (Table 1) as follows:

1. User’s previous PTC knowledge (Q1)
2. User’s previous experience with simulators (Q2)
3. User’s confidence to perform a PTC before simulation training (Q3)
4. User’s confidence to perform a PTC after simulation training (Q4)
5. User’s perception of the simulator system (Q5, Q6)
6. General user evaluation (Q7, Q8)

**Table 1:**
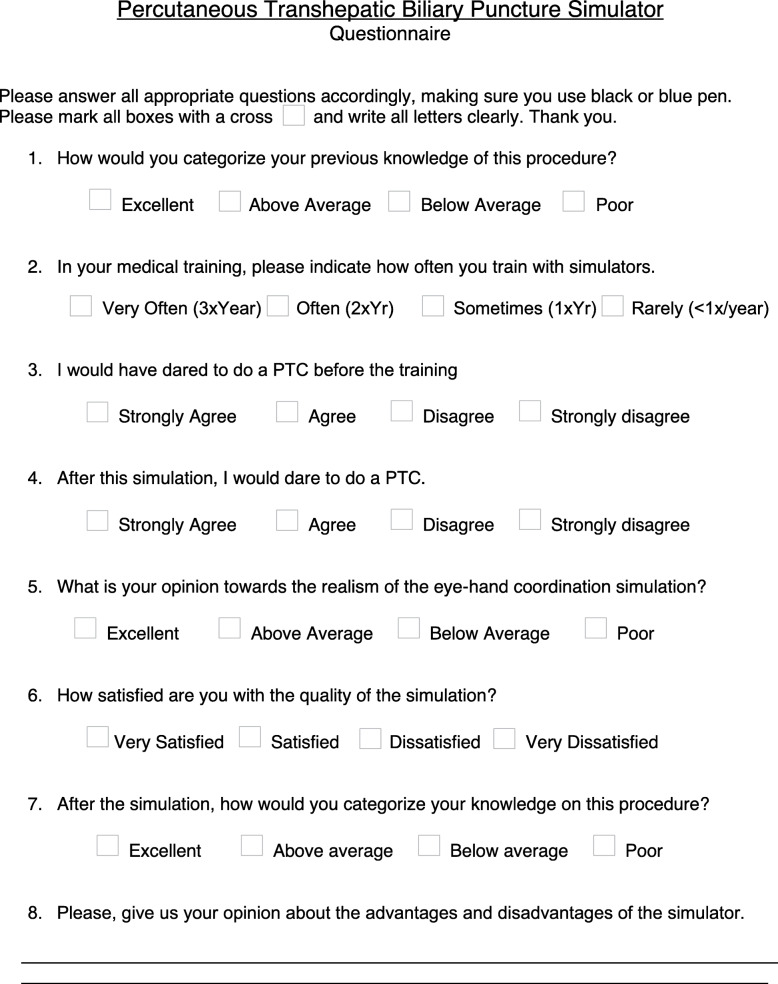
Questionnaire

## Results

Sixty radiology residents (*n* = 60) were included to test our PTC simulator. All participants completed all questions, the first three questions were completed before the tutorial explanation, and the next five questions were completed after the training on the PTC simulator.

### User’s previous PTC knowledge

Thirty (*n* = 30; 50%) participants categorized their previous knowledge of PTC as “poor,” twenty-six (*n* = 26; 43%) as “below average,” and four (*n* = 4; 7%) as “above average.”

### User’s previous experience with simulators

Forty (*n* = 40; 67%) participants indicated that they have trained with medical simulators “rarely (< 1 time per year),” fifteen (*n* = 15; 25%) answered “sometimes (1 time per year),” and five (*n* = 5; 8%) indicated “often (2 times per year).”

### User’s willingness to perform a PTC procedure before simulation training

Before the training, twenty-eight (*n* = 28; 47%) participants answered that they would “strongly disagree” to perform a PTC procedure and seventeen (*n* = 17; 28%) marked “disagree,” while eight (*n* = 8; 13%) and seven (*n* = 7; 12%) determined that they would “agree” and “strongly agree,” respectively.

### User’s willingness to perform a PTC procedure after simulation training

After one session of training on the PTC simulator, twenty-six (*n* = 26; 43%) participants determined that they would “agree” to perform a PTC, twenty-five (*n* = 25; 42%) answered that they would “disagree,” five (*n* = 5; 8%) marked “strongly agree,” and four (*n* = 4; 7%) marked “strongly disagree.”

### Realism of the eye-hand coordination simulation

Forty-four (*n* = 44; 73%) of the participants designated the realism of the eye-hand coordination simulation as “above average,” fifteen (*n* = 15; 25%) marked it as “excellent,” and one (*n* = 1; 2%) marked it as “below average.”

### User satisfaction related to the PTC simulator

Twenty-six (*n* = 26; 43%) participants declared that they were “very satisfied” with the quality of the PTC simulator, thirty-two (*n* = 32; 53%) marked “satisfied,” and two (*n* = 2; 3%) were “dissatisfied.”

### User PTC knowledge after simulator training

After the use of the PTC simulator, forty-six (*n* = 46; 77%) participants considered their level of knowledge of this procedure as “above average,” nine (*n* = 9; 15%) considered it to be “below average,” four (*n* = 4; 7%) marked “excellent,” and one (*n* = 1; 2%) designated his knowledge as “poor.”

### User open opinion toward the PTC Simulator

The most common advantage that participants declared was “the PTC simulator gives the opportunity to understand how to control a needle in a 2D perspective and train eye to hand coordination,” and the most common disadvantage declared was “the camera is not able to angulate, so there is only one projection.”

### Simulator training results

All participants (*n* = 60; 100%) were able to troll a cord by themselves, with an average time of 1:34 min. Twenty-six (*n* = 26; 43%) trolled the cord before the first minute, sixteen (*n* = 16; 27%) before the second minute, thirteen (*n* = 13; 22%) before the third minute, and five (*n* = 5; 8%) before the fourth minute. Only one (*n* = 1; 2%) participant punctured the simulated pleura. The number of punctures and the distance away from the target were not quantified due to it would cause undesired stress on the participant and interrupt the learning process.

## Discussion

IR procedures rely on the operator’s sense of touch and the use of medical imaging to guide a needle toward a specific, anatomical, or pathological structure. For all needle placements, getting the needle from point A to B is a technical requirement. In biliary system interventions, and specifically PTC, the risk of vascular damage and internal bleeding is proportional to the number of punctures performed during the procedure and depends as well on the correct location of the target point. However, bleeding complications are not the only risks during PTC. Injuries to other structures, such as the lung, intestine, or vessels outside the liver (e.g., intercostal vessels), represent potential sources of complications when the wrong puncture technique is used [8, 9].

Challenging procedures require additional training, and this can be achieved using simulators. These devices enable novice operators to train in a range of case scenarios and allow prediction and measurement of procedural outcomes before performing the procedure on real patients [10]. Simulation is of vital importance for procedural and clinical IR training and is increasingly demanded by our evolving healthcare environment of value-based care and persistently high medical error rates [11, 12].

In our study, we observed that 93% of the participants considered their technical knowledge about PTC procedures to be poor. This is an indicator to us that interventional procedures should be better promoted in this group of young specialists during their residence time, in a similar way to that now used for computer tomography or magnetic resonance disciplines.

**Fig. 4:**
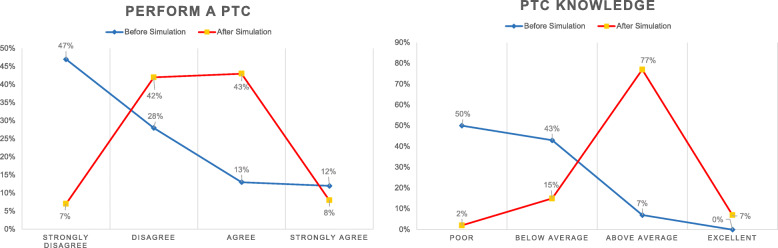
Graphics illustrating the increase of confidence and subjective PTC knowledge after the simulation

Overall, 75% of the participants initially showed a strong unwillingness to perform a PTC in a real patient, even under tutorial surveillance. However, this number decreased to 15% after training in our simulator (Fig. 4). This finding does not indicate that the student is necessarily prepared to perform a real PTC procedure and can be explained as learner engagement and suspension of disbelief (improved self-confidence) phenomenon’s, this means that after using the simulator the participant becomes more interested in testing his skills and putting his newly acquired knowledge into practice [12]. However, transfer to real-life practice could not be demonstrated in our study because of an ethical and local educational program restriction; further research is needed to test the transfer of knowledge to the clinical level. The results regarding the eye-hand coordination were excellent (98%). We took particular care to make the simulator with a physical resemblance to a real liver, while at the same time allowing the participants to understand the importance of the three-dimensional orientation (ventral, dorsal, cranial, caudal, medial, and lateral).

Simulators are now increasingly available for medical training; however, some of them are expensive or require complex installation and software. In our study, we demonstrated the possibility of creating efficient simulators with good acceptance and good educational results at a low cost and without the necessity of complex software. Additional advantages of our PTC simulator are its easy transportation, fast installation, low weight (1.5 kg), and zero X-ray exposure. Disadvantages are the inability to rotate the camera in the same way as an angiography C-arm and the necessity of holding the system during the punctures.

## Conclusion

The main focus of this investigation was to present a simulator that satisfies all the initial PTC requirements, including anatomical appearance, modularity, reusability, minimal cost, and the ability to discover technical errors in real time. This PTC simulator provides radiology residents with the ability to learn and rationalize the concepts and workflow of a standard PTC procedure in a safe and controllable environment. This proposed simulator can be a functional tool for improving user’s confidence and competence while reaching procedural and non-procedural IR skills related to the liver. We believe that this simulator is ideal for developing radiology resident understanding of the technical challenges and the first steps of a PTC.

### Limitations of our model

No simulation of breathing movement and more complex situations, such as non-dilated biliary tree or accidental puncture of hepatic vessels, were done with this model; moreover, no ultrasound guidance was used for simulations shown in this study.

## Data Availability

Not applicable

## Abbreviations

PTC: Percutaneous transhepatic cholangiography
IR: Interventional radiology
Q: Question
